# Molecular Analysis of miR-155 and MIR155HG Mutations in Conjunction with TLR4 Variants in Inflammatory Joint Disease

**DOI:** 10.3390/cimb48040400

**Published:** 2026-04-14

**Authors:** Wissam Albeer Nooh, Raya Kh. Yashooa, Abdullah W. Khaleel, Treska S. Hassan, Shawnim M. Maaruf, Safa M. Salim, Abd Al-Bar Al-Farha, Suhad A. Mustafa, Dara K. Mohammad

**Affiliations:** 1 Department of Biology, College of Education for Pure Sciences, University of Al-Hamdaniya, Mosul 41002, Iraq; wissamalbeer@uohamdaniya.edu.iq (W.A.N.); raya.yashooa@uohamdaniya.edu.iq (R.K.Y.); 2 Cancer Research and Hereditary Blood Diseases, Technical Research Center, Northern Technical University, Mosul 41001, Iraq; abdullah.khaleel17@ntu.edu.iq (A.W.K.); safasalim244@ntu.edu.iq (S.M.S.); 3 General Directorate of Scientific Research Center, Salahaddin University-Erbil, Erbil 44001, Iraq; treska.hasan@su.edu.krd (T.S.H.); shawnim.maaruf@su.edu.krd (S.M.M.); suhad.mustafa@su.edu.krd (S.A.M.); 4 Department of Biotechnology and Food Sciences, Technical Agricultural College-Mosul, Northern Technical University, Mosul 41001, Iraq; dr.abdalbar@ntu.edu.iq; 5 Center for Hematology and Regenerative Medicine (HERM), Department of Medicine Huddinge, Karolinska Institutet, SE-141 83 Stockholm, Sweden; 6 College of Agricultural Engineering Sciences, Salahaddin University-Erbil, Erbil 44001, Iraq

**Keywords:** miR155, MIRAHG155, TLR4, osteoarthritis, inflammatory joint diseases

## Abstract

Inflammatory joint diseases, including osteoarthritis, are multifactorial disorders in which dysregulated innate immune signaling and non-coding RNA (ncRNA)-mediated regulation of gene expression play essential roles. MicroRNA-155 (miR-155), its host gene MIR155HG, and Toll-like receptor 4 (TLR4) form a tightly linked inflammatory signaling axis, yet their combined genetic variability in chronic joint inflammation remains insufficiently characterized. The aim of this study was to investigate genetic variants in MIR155HG exon 3, mature miR-155, and TLR4 exon 3 and assess their potential synergistic role in chronic inflammatory joint disease. A case–control study was conducted with 100 cases (50 osteoarthritis patients and 50 matched healthy controls). Genomic DNA was analysed using polymerase chain reaction (PCR) and Sanger sequencing. Variant alleles and genotypes were identified, and their allele frequencies and genotypes were calculated using Mutation Surveyor. Detected variants were compared with public databases, and in silico tools were used to estimate the structural impact of TLR4 missense mutations. Sixteen heterozygous variants were identified in MIR155HG exon 3, most of them novel and population-specific. Interestingly, the highest variant frequencies for MIR155HG exon 3 were observed at positions 12448G>GC and 12481T>TA (both 64.3%), followed by 12442T>TC (57.1%). Additionally, two novel variants were detected in the miR-155 gene (chr21:29,694,314 G>A and chr21:29,646,351 T>C), each present at an allele frequency of 7.1% and absent from current external variant databases. Moreover, two rare TLR4 exon-3 variants were identified; a synonymous variant, c.147C>A (Pro49Pro; rs375037549), and a missense mutation, c.148G>A (Asp50Asn; rs776561489). Notably, in silico analyses and molecular dynamic simulations indicated that the Asp50Asn (D50N) substitution destabilizes the TLR4 protein. Conclusion: Concurrent variants in MIR155HG, miR-155, and TLR4 suggest a convergent regulatory molecular axis that may contribute to disease susceptibility and inflammatory progression.

## 1. Introduction

Inflammatory joint disorders, including osteoarthritis (OA) and rheumatoid arthritis, lead to a decline in function and reduced quality of life [1]. At present, there is no effective medication used to inhibit the onset and disease progression, and the OA severity often intensifies with age [2]. It is estimated that 18 percent of females and 9.6 percent of males globally, above the age of 60 years, experience OA symptoms [3]. Inflammatory joint disorders feature cartilage degeneration, subchondral bone pathology, and concurrent synovitis, while the mechanisms underlying OA pathogenesis are not fully understood and are believed to be multifactorial [4]. MIR155 Host gene (MIR155HG), a long non-coding RNA host gene with pro-inflammatory capabilities, was once thought to be involved in the human immune system response [5]. It is also known as the B-cell integration cluster, which is defined by a gene on chromosome 21q21 [6].

A microRNA (miR) is a small non-coding RNA (sncRNA) that plays a significant role in regulating gene expression and is dysregulated in numerous pathological conditions [7,8]. MIR155HG transforms into miR-155, the miR-155HG, and genetic variants in MIR155HG have the potential to affect miR-155 production [9]. MIR155HG has been linked with various inflammatory diseases [10,11]. Chronic immune activation, cytokine secretion, and escalating tissue damage are hallmarks of inflammatory joint diseases such as rheumatoid arthritis (RA) and OA [12,13]. miR-155 is one of the microRNAs that is most consistently elevated in inflamed synovial tissues. It facilitates pro-inflammatory macrophage and T-cell responses [14]. The host gene MIR155HG is rigorously regulated. This gene encodes the primary transcript required for miR-155 synthesis [15]. Thus, variations in MIR155HG may directly affect miR-155 levels, leading to individual differences in inflammatory outcomes [16]. miR-155 gene expression has been linked to numerous chronic and severe inflammatory diseases and autoimmune disorders, including RA, inflammatory bowel disease (IBD), atherosclerosis, and systemic lupus erythematosus (SLE) [17,18].

Toll-like receptor 4 (TLR4) is a transmembrane protein essential for recognizing pathogens and initiating inflammatory signaling pathways, as it regulates the activation of antigen-presenting cells and the production of critical cytokines, thereby connecting innate and adaptive immunity. TLR4 is a crucial pattern recognition receptor that activates a defensive immune response and initiates inflammatory cascades via the MAPK and NF-κB signaling pathways [19,20]. In osteoarthritis, TLR activation induces a pro-inflammatory, catabolic state that subsequently alters the anatomical and physiological functions of the entire joint [21]. Prior work has demonstrated an association between TLR4 and the occurrence of inflammatory joint disease [22,23]. The activation of TLR4 induces the production of miR-155, which then regulates negative feedback molecules within the TLR4 pathway, establishing a regulatory loop between the two entities [24]. Genetic alterations in TLR4, particularly within functional domains such as exon 3, may disrupt receptor signaling and alter miR-155 activity.

The present study concurrently investigates and identifies novel genetic mutation variants in miR-155, MIR155HG, and TLR4 in the exonic regions, and demonstrates whether genetic alterations could stimulate at both loci and may provoke aberrant inflammatory pathways and signaling, from non-coding RNA (ncRNA) regulation to innate immune receptor function, and illuminates a potentiating pathway influencing chronic inflammation on joint disease. This integrative framework clarifies the combination of host gene genetic variants, dysregulation of microRNA, and receptor signaling anomalies in the etiology of chronic joint disease. This study sought to clarify the molecular relationship between miRNA-mediated regulation, host gene activity, and innate immune receptor signaling in the context of chronic joint inflammation.

## 2. Materials and Methods

### 2.1. Ethical Approval of Research

This study adhered to the principles of the Declaration of Helsinki and received approval from the Human Ethics Committee (HEC) at Salahaddin University-Erbil/College of Science, Biology Department (Ref. No. 45/275, 22 February 2021). Before participating in the study, all volunteers signed a document confirming their understanding of the procedures.

### 2.2. Sample Collection

This case–control study, conducted at a hospital, examined osteoarthritis (OA) in 100 participants matched by gender from Erbil city. The study comprised 50 patients with clinically diagnosed osteoarthritis and 50 healthy controls. Patients were identified in orthopedic clinics and private clinic institutions within the region. Eligibility for the OA study required participants to have a clinically confirmed diagnosis, as confirmed by X-rays, be at least 40 years of age, and have knee discomfort for over 6 months, regardless of gender. We obtained control participants from carers at the same facilities and matched them to the OA group by age and gender. Clinical assessment verified that the controls had no history of osteoarthritis, joint pain, or inflammatory arthritis. Individuals in both cohorts were ineligible if they had autoimmune diseases, such as SLE, antiphospholipid syndrome, or RA, or if they had experienced a fracture or surgery within 6 months of enrolment in the study. A total of 5 mL of peripheral blood samples was collected from all participants utilizing an anti-coagulation tube, EDTA-K3, to prevent blood coagulation. The blood samples were immediately stored at −80 °C until they could be processed for molecular analysis, including genomic DNA extraction and PCR amplification.

### 2.3. Extraction of DNA

Human genomic DNA was isolated from a lymphocyte sample. A spin-column kit from Jena Bioscience (Jena, Thuringia, Germany, Cat. No. PP-237S) was used to extract DNA according to the manufacturer’s guidelines. We used a NanoDrop™ spectrophotometer (ThermoFisher Scientific, Waltham, MA, USA) to meticulously assess the quality and purity of the isolated genomic DNA. The A260/A280 ratio indicated that purity consistently ranged from 1.7 to 2.0, while DNA concentrations varied from 60 to 320 ng/µL. Electrophoresis carried out on a 1.5% agarose gel provided further evidence that the genomic DNA remained intact. The extracted genomic DNA was stored at −80 °C until use for molecular and genetic investigations.

### 2.4. MIR155, M155HG and TLR4 Mutation and Genotyping Analysis

The study investigated two genes: MIR155, a portion of MIR155HG, and TLR4 exon 3. The purified genomic DNA was amplified for each genetic variant utilizing PCR (M1000-G PCR, Stockholm, Sweden). The primers were designed by (http://primer1.soton.ac.uk/primer1.html), accessed on 9 January 2025, and manufactured by (Macrogen, Seoul, Republic of Korea); the primers are listed below. The primer designed for MIR155 to amplify genomic DNA fragments extended 400 bp upstream and 400 bp downstream of MIR155 gene to ensure coverage of the target region of exon; the sequencing results revealed that the PCR products included a portion of MIR155HG exon 3, which was subsequently analyzed for potential variants. MIR155 Forward primer: AATTACTTCCAAGGATTTAATGAGCTCC, reverse primer: ACATCCCAGTGACCAG-ATTATGATTAAC; TLR4 Exon3 Forward primer: GATGG-ATGGATATATGGATGG-AAGGATG, reverse primer: CAAACCAAGCTTTCCAGTCCAATAATGA. The master mix, ready-to-use (ADDBIO Inc., Daejeon, Republic of Korea, Cat. No. 36101), consisting of Taq DNA polymerase, KCl, dNTPs, and reaction buffer, has been utilized. For primer optimization, a gradient PCR was performed over 10 temperatures ranging from 55 to 65 °C. The bands appeared at 59 °C for all polymorphisms (MIR155, MIR155HG exon 3, TLR4 exon 3).

The conditions of the PCR thermocycle consist of the following: initial denaturation for 5 min at 95 °C, followed by 40 cycles of denaturation at 95 °C for 20 s, annealing at 59 °C for 45 s, and extension at 72 °C for 20 s, then final extension at 72 °C for 5 min. The PCR was performed in a 20 μL reaction volume containing 10 μL master mix, 3 μL of genomic DNA, 1 μL each of forward and reverse primers, and 5 μL ddH_2_O. Electrophoresis (Cleaver, London, UK) was utilized to analyses the amplicons generated by the post-PCR. A 2% agarose gel was utilized to separate the PCR products, alongside a 100 bp DNA ladder (ADDBIO Inc., Daejeon, Republic of Korea) for comparison; 435 bp for MIR155; and 413 bp for TLR4 exon3; the reference segment in the DNA ladder corresponded to MIR155, MIR155HG exon 3, and TLR4 exon 3 gene loci. Before casting, the agarose gel was stained with a safe dye (ADDBIO Inc., Daejeon, Republic of Korea). A gel documentation system (Proxima 2500) from Biosave Ltd., Harrogate, UK) was employed to visualize the amplified DNA bands.

### 2.5. Sample Sequencing

To identify and characterise genetic variations and polymorphisms in the TLR4 and MIR155 genes in both the study and control groups, PCR products were prepared for Sanger sequencing. Macrogen Inc. (Seoul, Republic of Korea) sequenced approximately 40 μL of each PCR amplicon, together with the forward primer specific to the target genes. An automated (3730xl DNA Analyzer) (Applied Biosystems; Thermo Fisher Scientific, Inc., Waltham, MA, USA) was utilized.

### 2.6. Bioinformatic Analysis

We used Mutation Surveyor software (version 5.1.2; Soft Genetics, State College, PA, USA) to analyze Sanger sequencing data and compare it with established variant databases, such as gnomAD, to ensure accurate variant annotation and assess clinical relevance. The software displays the results in hierarchical file tree format and with a comprehensive Output Report Table (ORT). The ORT displays all identified nucleotide alterations, associates them with their chromatogram traces, and presents their quality scores. Peak signal analysis, base-calling confidence metrics, and Phred quality scores collectively ensure high variant accuracy inside the ORT, hence enhancing the reliability of mutation identification. These may consist of sample-specific mutation call files or comprehensive variant lists for all examined areas. The Bentley report was utilised to assess genotype-based variants, providing additional insights into zygosity and allelic distribution of the identified variants. Data from gnomAD enhance the study’s utility by distinguishing pathogenic or suspected pathogenic variants from benign polymorphisms and by examining their population frequency, clinical significance, and previously established associations.

### 2.7. Statistical Analysis

The statistical analyses were conducted using GraphPad Prism (version 8.0.1, San Diego, CA, USA) and IBM SPSS (version 25, Chicago, IL, USA). The statistical analyses were conducted using GraphPad Prism (version 8.0.1, San Diego, CA, USA) and IBM SPSS (version 25, Chicago, IL, USA). The *t*-test (Mann–Whitney test) and Chi-square test were used to analyse patients’ characteristics; *p*-values < 0.05 were considered significant. The genetic data were analysed utilizing Mutation Surveyor software (version 5.1.2; SoftGenetics LLC., State College, PA, USA) to analyze sequencing data. This software identifies nucleotide variations by automatically aligning sample sequences to a reference sequence and using chromatogram peak analysis to evaluate variant calls. It applies an anti-correlation algorithm to compare Sanger sequencing chromatograms from each patient sample against a (Gene Back) reference sequence to detect and identify nucleotide variations. After aligning the reference and sample traces, the software analyses variations in shape, peak height, and spacing. For each identified variant, the mutation software calculates the variant relative contribution and the reference signal by determining the peak intensities in the chromatogram, allowing the calculation of the variant percentage (allelic ratio) as the proportion of the variant peak relative to the total signal at that position. This quantitative measurement can classify genotypes into heterozygous, homozygous reference, or homozygous variant states and can detect low variant frequencies, such as mosaicism, when peaks are available. As follows, the assignment genotype frequencies were estimated as the ratio of each genotype across all cases, and the allele frequencies were derived using standard formulas based on genotype counts.

## 3. Results

### 3.1. Characteristics of the Patients

A total of 100 subjects participated in the current study, including 50 osteoarthritis (OA) cases and 50 non-osteoarthritis controls. Table 1 summarizes the clinical features and demographic characteristics of patients’ cohorts and the healthy group. The mean age and sex of OA cases and healthy controls did not differ significantly between the two groups. Furthermore, the statistical analysis confirmed significant differences in body mass index (BMI) between the two cohorts. However, our results detected no significant differences in vitamin D and calcium levels between the two cohorts.

### 3.2. Mutation Analysis

Targeted sequencing of MIR155HG exon 3 in patients with chronic inflammatory joint disease, followed by Mutation Surveyor analysis, identified multiple single-nucleotide substitutions concentrated within exon 3. Sixteen distinct single-nucleotide variations (SNVs) were identified in the exon-3 area at MIR155HG chromosome 21, all in heterozygous form, and all constituted single-base substitutions (no frameshift insertions or deletions were detected). The variant caller’s consequence annotation classified most modifications as non_coding_transcript_exon_variant, indicating their presence in the MIR155HG transcript exon. The variant percentage in the study group differed by location. The proportion of identified variant alleles ranged from approximately 7.1% for some low-frequency SNVs to 64.3% for the predominant site, with several intermediate percentages between approximately 14% and 38% (see Table 2 for specific positions and variant percentages). The highest percentages were at chromosome positions 21:26946404 12448G>GC and 21:26946437 12481T>TA (both 64.3%), and 21:26946398 12442T>TC was 57.1%.

### 3.3. Identification of Novel Variants in miR-155 and TLR4

Mutation analysis of DNA from patients with chronic inflammatory joint disease revealed two unique heterozygous single-nucleotide variations in the miR-155 gene (chr21:29,694,314 G>A and chr21:29,646,351 T>C) (Table 3). Both variants are located in regions expected to affect the precursor miRNA configuration and potentially the production of mature miR-155. The allele frequency in the study group was 7.1%, and their absence in current external variant databases indicates that these are unique mature miRNA variants.

Interestingly, exon 3 of the TLR4 gene was sequenced in patients with chronic inflammatory joint disease, revealing two heterozygous nucleotide alterations. The initial alteration, c.147C>A (Pro49Pro), is a synonymous mutation (codon CCC → CCA) that does not alter the amino acid sequence. This was identified as a rare SNP in dbSNP (rs375037549). The second variant, c.148G>A (Asp50Asn), is a missense mutation (codon GAC → AAC) that alters aspartic acid to asparagine (rs776561489) (Table 4). This variation is located in the extracellular domain of TLR4, which influences ligand recognition and receptor signaling. It is currently catalogued in population databases as a rare allele with no established disease association. No further exonic mutations or indels were identified in this region, and both alterations were detected in high-quality sequencing reads. These alleles were confirmed to be unique and rare by cross-referencing with gnomAD, suggesting possible inclusion as underrepresented variants in the population under study.

### 3.4. Genotype Analysis

The Mutation Surveyor program analysis has revealed genotype frequencies of single-nucleotide variants (SNVs) in TLR4 exon 3 and the miR-155 gene in people with chronic joint inflammation and disease. Two variant loci were identified for the TLR4 gene: 4942 C>CA and 4943 G>GA. At position 4942, the heterozygous genotype CA was observed in 14.3% of cases, while the wild-type homozygous genotype CC was predominant at 85.7%. The heterozygous GA genotype was identified in only 3.6% of the 4943 locations, whereas the GG genotype was present in 96.4% of the cohort (Table 5). Additionally, two distinct variant sites were identified in the miR-155 gene. The 12358G>GA mutation displayed a similar distribution, with the GA genotype present in 3.6% of subjects and the homozygous GG genotype predominating at 96.4%. The 12395T>TC variant had a TC genotype frequency of 3.6%, whereas the TT genotype was predominant at 96.4% (Table 5).

Moreover, targeted sequencing of MIR155HG exon 3 in patients with chronic inflammatory joint disease identified multiple single-nucleotide variants (SNV) with distinct genotype frequency patterns. The homozygous wild-type genotype was predominant (96.4%) at multiple variant loci, including 12264A>AG, 12300T>TC, 12310G>GA, 12314A>AT, and 12434C>CG (Table 6). The heterozygous variants were seen at a low frequency of 3.6%. The 12408T>TG variant displayed the TG genotype in 7.1% of individuals, whereas the TT genotype remained the predominant form at 92.9%. In contrast, considerable heterozygosity was observed at multiple loci, including 12442T>TC (TC: 28.6%), 12448G>GC (GC: 32.1%), and 12471G>GC (GC: 82.1%) (Table 6), indicating that these loci may serve as mutation hotspots within exon 3 of MIR155HG. Moderate frequencies of heterozygous genotypes were seen at the following locations: 12481T>TA (32.1%), 12511T>TG (11.5%), 12514T>TA (11.5%), 12515C>CG (3.6%), 12518T>TA (7.7%), 12523T>TC (19.2%), and 12543T>TG (10.0%) (Table 6). The data revealed a mutation landscape in MIR155H and elevated heterozygous variants in some regions that could influence miR-155 expression and its contribution to the pathogenesis of inflammatory joint disease.

### 3.5. Mutation Summary Retrieval from the Database

The analysis of the gnomAD v4.1.0 database was previously conducted to explain the mutational profiles at three specific loci: TLR4, MIR155HG, and miR-155. The results show a genetic variation scenario that aligned with the functional classification of each gene.

The protein-coding TLR4 gene contains 1847 distinct variants, with missense variants (1000) accounting for over 50% of all polymorphisms. Subsequently, there are 412 synonymous variations and 195 intron variants (Table 7). This pattern is characteristic of a protein-coding gene subjected to evolutionary pressure. A significant number of frameshift (55) and stop-gained (47) variations suggest the presence of several loss-of-function alleles that may impact innate immunity.

A total of 511 variants were identified for MIR155HG, the long non-coding RNA that serves as the host for miR-155 (Table 8). Most of the mutational landscape consists of non-coding transcript exon variants (412) and intron variants (84). This profile indicates that the gene does not encode proteins and that polymorphisms mainly alter the structure, stability, or processing of the transcript rather than the amino acid sequence. Alterations in splice regions (splice region: 8, splice acceptor: 5, splice donor: 2) may influence the synthesis and functionality of the lncRNA. miR-155 was involved in 15 distinct variants. The absence of variation in this gene indicates a functional limitation and its significance in regulatory networks. The variants are primarily categorized into two groups: mature microRNA variants (10) and non-coding transcript exon variants (5). Polymorphisms in the mature microRNA sequence are significant because they may alter target mRNA recognition, potentially leading to abnormal gene expression and diseases such as cancer or inflammation.

### 3.6. Prediction of Structural Stability for TLR4 D50N Mutation

**Figure 1:**
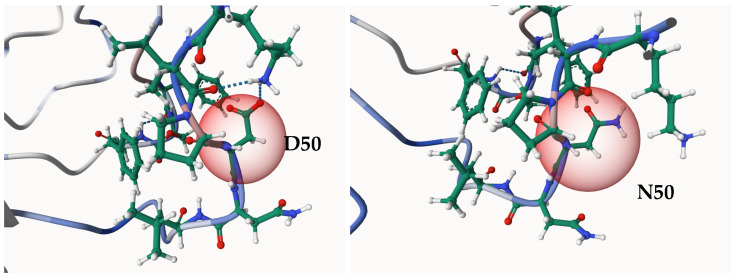
TLR4 D50N mutation found in the 3D structure by the Mutation Explorer. Surface and backbone models of human TLR4 are presented to show the location of the D50N mutation. The Aspartate (D50) in position 50 is replaced with Asparagine (N50) in the mutant. The replaced residue is shown in a different color and magnified. It is present in the receptor’s N-terminal extracellular domain, which is involved in recognition and folding. The results indicate that the D50N mutation does not impact the course of mutagenesis. This observation indicates the environment of residue 50 and the effects of the D50N mutation on interactions with side chains and protein stability. This figure was generated using the Mutation Explorer Webserver (https://mutationexplorer.vda-group.de/mutation_explorer/), accessed on 11 March 2026, with default settings.

The effect of the Aspartic acid (D) residue to Asparagine (N) substitution at position 50 (D50N), which lies within a Leucine Rich Repeat (LRR) domain, was assessed using three distinct computational methods integrated within the MuPro platform: a Support Vector Machine (SVM) model for quantitative Delta\Delta G (ΔΔG) prediction, and two separate models (SVM and Neural Network) for qualitative prediction of the sign of the energy change [25]. Interestingly, all three approaches indicate that the D50N mutation negatively affects the stability of the TLR4 protein. In protein stability prediction, a negative ΔΔG value means that the mutant protein is less stable than the wild-type protein. The relatively large value indicates that the thermodynamic stability of the TLR4 protein is greatly reduced. Moreover, the directional predictions for the energy change also support this view. The SVM and Neural Network models predicted a destabilizing effect with confidence scores of −1.00 and −0.76, respectively. The platform uses the negative sign of the confidence score to denote a destabilizing effect. The high absolute values of these scores increase the reliability of the predicted destabilization (Table 9). Next, the stability prediction of the substitution at position 50 (D50N) on the 3D conformational change in TLR4 was analysed using the Mutation-Explorer platform [26]. This analysis is crucial for understanding the mutation’s potential pathogenicity, as reduced protein stability may lead to misfolding, aggregation, or accelerated degradation (Figure 1).

**Figure 2:**
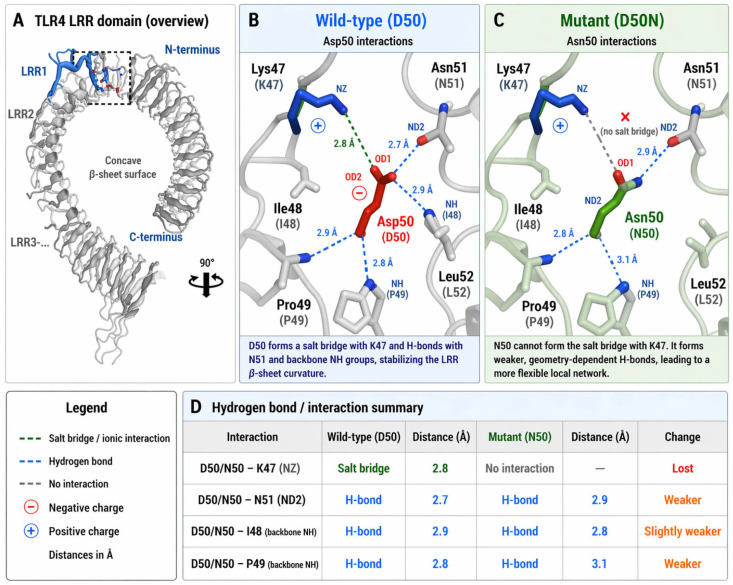
Hydrogen bonding interaction comparison between wild-type Aspartate (D50) and Asparagine (N50) within the TLR4 (LRR) domain. (**A**) TLR4 LRR domain and N-terminus overview of concaveBetasheet surface, where D50 (Wt) or N50 (Mut) is located. The area inside the box shows the local structural environment that was studied using Molecular Dynamics (MD) Simulations. (**B**) In the wild-type TLR LRR domain, D50 makes a salt bridge with K47 that is about 2.8 Å away and takes part in several hydrogen bonds, such as those with N51 (about 2.7 Å), the backbone of I48 (about 2.9 Å), and P49 (about 2.8 Å). These interactions help maintain the β-sheet curvature as stable in this region. (**C**) In the mutated TLR LRR domain (N50), the substitution diminishes the salt bridge with K47, and the residue forms weaker hydrogen bonds with N51 (~2.9 Å), I48 (~2.8 Å), and P49 (~3.1 Å). Such changes suggest that the geometry was changed and the strength of interaction weakened. (**D**) A summary table of the bond formations and their distances (Å), elucidating the differences in the hydrogen bond network and the electrostatic interaction between wild-type and mutant TLR4.

Consistent with the predicted structural effects described above, to understand how the D50N change in Toll-like receptor 4 affected its structure, the detailed hydrogen-bonding network of residue 50 and its interactions in the N-terminal LRR region were analysed using Visual Molecular Dynamics (VMD) and HBPLUS v.3.06 (Figure 2A). In the wild-type configuration, Asp50 (D50) participates in multiple stabilizing interactions and primarily forms a strong electrostatic interaction with Lys47 (K47), creating a salt bridge at about 2.8 Å. On the local interaction side, this bridge is a significant component of the interaction network, providing it with stability. Additionally, Aspartate 50 (D50), through its negatively charged carboxylate group, forms hydrogen bonds with Asn51 (N51) (2.7 Å) and the backbone amide groups of Ile48 (I48) (2.9 Å) and Pro49 (P49) (2.8 Å) (Figure 2B). Collectively, these networks can maintain the structural integrity and curvature of the concave β-sheet in the TLR4 LRR domain.

Conversely, when D50 was mutated to N50, the salt-bridge with K47 was disrupted. Interestingly, the N50 was still capable of forming hydrogen-bonds, although they were weaker and had a different shape than in the wild type. Also, the N50 residue could form longer hydrogen-bonds with N51 and P49 at 2.9 Å and 3.1 Å, respectively. Additionally, the hydrogen-bond distance between N50 and I48 (2.8 Å) was a bit less favorable than in wild type (Figure 2C). Importantly, these relative variations in bond distances and the new interaction geometry are also consistent with the anticipated drop in local electrostatic strength and the slight destabilisation (Figure 2D). These findings demonstrate that the D50 residue might play an important role as an electrostatic anchor in the LRR domain. Its substitution with asparagine (N) could destabilize the local structural environment, which is either subtle to detect but functionally relevant, or alter receptor conformational dynamics and consequent TLR4 signal transduction.

## 4. Discussion

This study clarifies a novel insight into the molecular and genetic underpinnings of chronic inflammatory joint disease by simultaneously investigating MIR155HG exon 3, TLR4 exon 3, and the regulatory role of their shared effector, miR-155. Our results revealed that MIR155HG harbors several substitutions, predominantly in exon 3, many of which are absent from external databases and exhibit diverse allele frequencies across the population. Two rare TLR4 exon 3 variants were identified concurrently: a synonymous mutation (p. Pro49Pro, rs375037549) and a missense modification (p. Asp50Asn, rs776561489).

In addition to two novel mutations detected in the miR-155 gene, these data, in conjunction with existing knowledge of miR-155′s involvement in inflammation, substantiate the notion of a genetic mechanism influencing both innate immunity and inflammation in joint disease. While we detected variants across TLR4, MIR155HG, and miR-155, we confirm that our current findings do not straightforwardly describe functional coordination or interaction regulation within loci. Accordingly, interpret the co-occurrence of genetic variants across MIR155HG, miR-155, and TLR4 as biologically reasonable rather than as confirmed evidence of a convergent regulatory axis. These results and interpretations are supported by extensive evidence and previous published findings demonstrating the functional association of these loci. miR-155 is transcribed from the MIR155HHG host gene, and its promoter involves a substantiated NF-κB p50/p65 element responsive to inflammatory stimulation [16,27]. Moreover, MIR155HG encodes both IncRNA-155 and miR-155, which separately influence innate immune responses [28,29], confirming MIR155HG as a multi-regulatory functional immune locus. miR-155 is a characterized regulator of TLR4 initiated immune inflammatory signaling; TLR4 stimulation is within the most effective modulators of miR-155 expression, and miR-155 reciprocally potent TLR4 pathway components consisting of SOCS1 and TAB2, creating an accurately regulated feedback pathway [1,30]. This TLR4, NF-κB, miR-155 regulatory signaling network has also been identified in macrophages [31]. In addition, enhancers regulated by the BET bromodomain and NF-κB proteins drive miR-155 transcription, leading to downregulation of canonical inflammatory gene expression [32].

LncRNAs play crucial roles in cellular activities via several processes, including epigenetic modification, post-transcriptional processing, regulation, and translation [33]. LncRNAs are implicated in cell differentiation and activation, playing a crucial role in regulating the development and function of immune cells in both the innate and adaptive immune systems [34]. The recently identified variants in exon 3 of MIR155HG within this cohort may indicate regulatory hotspots. Despite being termed non-coding transcript exon variants, their proximity to pri-miRNA hairpin structures suggests they may influence Drosha/DGCR8 function or RNA stability. The population-specific enrichment of these variants, with allele frequencies of 64%, indicates their potential cumulative impact on disease susceptibility within this genetic framework, highlighting the importance of studying underrepresented populations in miRNA genetics. Significantly, previous studies found that the miRNA-155 precursor sequence, encoded by MIR155HG, furthermore functions as a lncRNA, designated lncRNA-155 [28]. miRNA-155 has been extensively identified as a major regulator of inflammatory responses [35,36]. MIR155HG may possess additional roles unrelated to the miR-155 synthesis. MIR155HG has been shown to function independently of miR-155, particularly during infection [32]. Genetic alterations to the host gene (MIR155HG) or the mature miR-155 sequence may significantly impact biogenesis, expression, and downstream signaling, hence altering the response of synovial tissues to inflammation. SNPs are the predominant form of genetic variation that influences disease risk via modulating the expression of associated genes.

The previous investigations have revealed an association between SNPs of MIR155HG and epilepsy [37], multiple sclerosis (MS) [38], osteonecrosis [39], atopic eczema [40], and various types of cancer [41]. In our study, we identified novel mutations that contribute to the development of inflammatory joint disease; however, few prior studies have investigated MIR155HG in this context. We anticipated that genetic diversity in the MIR155 host gene may affect the production of its transcriptional product, miR-155, thereby influencing its role in inflammatory diseases. Recent research on miRNAs in the immune system indicated that miR-155 is correlated with OA [42]. Furthermore, it has been documented that elevated miR-155-5p expression induces M1 polarization in the macrophage RAW264.7 cell line [43,44]. Reports indicate that miR-155-5p facilitates M1 macrophage polarization by targeting suppressor of cytokine signaling 1 (SOCS1), which modulates STAT3 and AKT signaling pathways [45]. miR-155-5p regulation may serve as a prospective therapeutic target for the treatment of knee osteoarthritis [46].

A previous study in chondrocytes found substantial upregulation of miR-155 expression in response to LPS. LPS induction consistently elevates miR-155 expression in chondrocytes [47]. A prior study has investigated the genetic polymorphism rs767649 (A/T) in the Promoter region connected with osteoarthritis cases; however, the analysis detected no correlation [48]. Additionally, an association between rs767649 (A/T) and multiple sclerosis has been demonstrated in an Egyptian cohort [49]. The miR-155 polymorphisms have been studied in various contexts, including inflammation, osteoarthritis, and rheumatoid arthritis [50,51].

In the arthritic joint, endogenous TLR ligands cause synovial monocytes and macrophages to express miR-155 more frequently, which in turn causes the pro-inflammatory TNF-α, cytokines, IL-1β, and IL-6 to be produced at higher levels [52], while SOCS1 and MyD88 are downregulated [53]. Additionally, the anti-inflammatory TLR suppressor SHIP-1 protein (SH2-containing inositol phosphatase-1) is also a key regulator of osteoblast proliferation and differentiation [54]. The identification of TLR4 exon 3 variations simultaneously introduces a receptor-level aspect to the regulatory loop. The missense Asp50Asn alteration transpires inside the extracellular leucine-rich repeat domain essential for ligand recognition. The structural similarity to the well-studied Asp299Gly and Thr399Ile polymorphisms, which have been shown to alter LPS responses, suggests that Asp50Asn may alter the receptor’s shape or ligand affinity, thereby altering NF-κB signaling [55,56]. The synonymous Pro49Pro variant cannot be considered functionally silent, as synonymous alterations may influence codon usage bias, mRNA secondary structure, or translation kinetics [57]. TLR4 is an essential component for the innate immune system that identifies lipopolysaccharide LP endotoxin from Gram-negative bacteria [58]. In recent investigations, TLRs have been identified as crucial elements in recognizing endogenous damage-associated molecular patterns (DAMPs) generated by locally injured cells and danger signals from other immune cells [59,60]. Upon activation, many intracellular signaling pathways are activated, potentially leading to enhanced cytokine expression. In rheumatoid arthritis, activation of TLR4 has been shown to increase levels of IL-1β, IL-6, TNF-α, IL-10, and IL-17 [61,62]. Moreover, inhibiting TLR4 appears to alleviate this reaction and has consequently been suggested as a potential therapeutic target in rheumatoid arthritis [63]. This suggests that TLR4 SNPs may influence disease severity in inflammatory joint disease by altering TLR4 function, TLR4 gene expression, or by acting in proximity to other deleterious gene mutations. Previous studies have identified that TLR4 can enhance the occurrence of osteoarthritis [48]. In conjunction with MIR155HG exon-3 alleles, these TLR4 variations may alter the setpoint of the TLR4•NF-κB•miR-155 pathway in joint tissues, providing a molecular rationale for their concurrent study in inflammatory joint disease [64].

The computational prediction of structural destabilization (ΔΔG = −0.65 kcal/mol) for the TLR4 D50N mutation suggests a potential loss-of-function phenotype. Generally, reduced structural stability predisposes a protein to misfolding, impaired trafficking, reduced ligand binding, defective dimerization, or increased degradation. Therefore, the predicted destabilization of TLR4 D50N suggests a likely loss-of-function phenotype, characterized by weakened or absent signaling in response to LPS. Such impairment would be expected to diminish inflammatory responses and may increase susceptibility to severe bacterial infections. Our findings from prediction stability analysis open avenues for further research. Empirical confirmation of the computational predictions could be achieved by experimental validation of the postulated effects of the D to N mutation on TLR4 stability employing experimental methods (circular dichroism spectroscopy or differential scanning calorimetry). Also, to validate this in a lab environment, a multi-step experimental plan would be necessary, shifting to functional rather than structural validation.

Altogether, these findings emphasize the need to combine computational predictions and experimental evidence to obtain a complete understanding of the consequences of genetic variation in immune-associated genes. Overall, the present data highlighted functional interactions with microRNA control (miR-155), host gene structure (MIR155HG), and innate immune receptor signaling (TLR4). This genetic modification mix can support long-term immune activation and joint tissue inflammation, providing new evidence on disease pathogenesis and potentially aiding in identifying molecular risk indicators.

## 5. Limitations

Some limitations must be acknowledged. The sample size is rather small, which is acceptable for an exploratory study; nonetheless, it complicates the identification of robust associations between genes and diseases and may inflate the effect size estimates of identified changes [65,66]. It is essential to recognize that variants observed at elevated rates in our cohort may represent common polymorphisms in the broader community rather than pathogenic variants. Benign polymorphisms are prevalent and not invariably detrimental [67,68]. We compared allele frequencies to population databases such as gnomAD; however, these comparisons are ineffective in small groups due to sampling variability. A variant’s rarity does not necessarily indicate pathogenicity, nor does its prevalence imply a functional involvement in complex polygenic illnesses. Therefore, our findings should be considered preliminary and indicative of potential hypotheses. Replication in larger, independent cohorts, ideally well-powered with sample sizes sufficient to detect modest genetic influences [63], together with functional validation studies, is essential to confirm genuine disease associations.

## 6. Conclusions

This paper presents an exploratory genetic investigation of three functionally associated loci, (MIR155HG, miR-155, and TLR4), in the context of chronic inflammatory joint disease. We detected several specific genetic variants in exon 3 of MIR155HG, unique mutations in the mature miR-155 region, and unusual exon 3 variants in TLR4, including a missense substitution predicted to destabilize receptor structure. Association analysis was conducted separately for each gene and did not reveal statistically significant risk associations; furthermore, the present research approach does not allow for inferences regarding the combined or interacting effects of variants across these loci. The independent detection of rare-variant enrichment in patients at three functionally linked loci in the TLR4/NF-κB inflammatory signaling pathway is significant and warrants further examination. Although these genes are involved in a well-defined regulatory circuit in which TLR4 activation induces NF-κB-dependent transcription of MIR155HG, resulting in the production of miR-155, which subsequently modulates components of the TLR4 pathway, the functional impact of the specific variants identified here on this pathway has yet to be determined. These findings enhance the understanding of population-specific genetic variability in critical immune regulatory genes. They may guide future research using larger cohorts, integrated multi-locus analyses, and functional validation to determine whether variants across these loci act independently or interact to affect inflammatory processes in osteoarthritis and associated joint disorders.

## Figures and Tables

**Table 1:** Clinical and demographic characteristics of participants.

Participant’s Characteristics	OA Patients n = 50	Normal Controls n = 50	*p*-Value
Age	49.3 ± 9.0 y	52.4 ± 8.7 y	0.070
Sex Male/Female	31/19	16/34	0.097
BMI kg/m^2^	25.5 ± 2.23	26.5 ± 2.90	0.045
Level of Vitamin D ng/mL	21.0 ± 8.1	18.7 ± 8.9	0.07
Calcium Level mg/dL	10.6 ± 11.7	10.9 ± 13.1	0.397
Previous Bone Fracture Yes/No	20/30	16/34	0.29

Mean ± SD, BMI = body mass index, y = year.

**Table 2:** Variant identification in MIR155HG Exon 3 gene in Inflammatory Joint Disease patient analyses with DNA variant mutation analysis.

Gene	Chromosome Position	Mutation	Mutation Genotype	Consequence	Heterozygous/Homozygous	Variants	Variant Percentages (%)	AA Change	External Database
MIR-155HG Exon 3	21:26946220	Substitution	A>AG	non_coding_transcript_exon_variant	Heterozygous	12264A>AG	7.1	non	Current study
	21:26946256	Substitution	T>TC	non_coding_transcript_exon_variant	Heterozygous	12300T>TC	7.1	non	rs780154516
	21:26946266	Substitution	G>GA	non_coding_transcript_exon_variant	Heterozygous	12310G>GA	7.1	non	Current study
	21:26946270	Substitution	T>TA	non_coding_transcript_exon_variant	Heterozygous	12314A>AT	7.1	Non	Current study
	21:26946364	Substitution	T>TG	non_coding_transcript_exon_variant	Heterozygous	12408T>TG	14.3	Non	Current study
	21:26946390	Substitution	C>CG	non_coding_transcript_exon_variant	Heterozygous	12434C>CG	7.1	Non	Current study
	21:26946398	Substitution	T>TC	non_coding_transcript_exon_variant	Heterozygous	12442T>TC	57.1	Non	Current study
	21:26946404	Substitution	G>GC	non_coding_transcript_exon_variant	Heterozygous	12448G>GC	64.3	Non	Current study
	21:26946427	Substitution	G>GC	non_coding_transcript_exon_variant	Heterozygous	12471G>GC	35.7	Non	Current study
	21:26946437	Substitution	T>TA	non_coding_transcript_exon_variant	Heterozygous	12481T>TA	64.3	Non	Current study
	21:26946467	Substitution	T>TG	non_coding_transcript_exon_variant	Heterozygous	12511T>TG	23.1	Non	Current study
	21:26946470	Substitution	T>TA	non_coding_transcript_exon_variant	Heterozygous	12514T>TA	23.1	Non	Current study
	21:26946471	Substitution	C>CG	non_coding_transcript_exon_variant	Heterozygous	12515C>CG	7.7	Non	Current study
	21:26946474	Substitution	T>TA	non_coding_transcript_exon_variant	Heterozygous	12518T>TA	15.4	Non	Current study
	21:26946479	Substitution	T>TC	non_coding_transcript_exon_variant	Heterozygous	12523T>TC	38.5	Non	Current study
	21:26946490	Substitution	T>TG	non_coding_transcript_exon_variant	Heterozygous	12534T>TG	20	Non	Current study

**Table 3:** Identification of variants in the miR-155 gene in Inflammatory Joint Disease patient analyses with DNA variant mutation analysis.

Gene	Chromosome Position	Mutation	Mutation Genotype	Consequence	Heterozygous/Homozygous	Variants	Variant Percentages (%)	External Database
MIR155	21:26946314	Substitution	G>GA	mature_miRAvariant	Heterozygous	12358G>GA	7.1	Current study
	21:29646351	Substitution	T>TC	mature_miRAvariant	Heterozygous	12395T>TC	7.1	Current study

**Table 4:** Detection of variants in the TLR 4 exon 3 gene in patients with Inflammatory Joint Disease analysed by DNA variant mutation analysis.

Gene	Chromosome Position	Mutation	Mutation Genotype	Consequence	Heterozygous/Homozygous	Variants	Codon	Protein	AA Change	External Database
TLR 4 Exon 3	9:120470894	Substitution	C>CA	Synonymous (silent) variant/somatic variant	Heterozygous	4942 C>CA	CCC/CCA	p. Pro49Pro	non	rs375037549 change to A
	9:120470895	Substitution	G>GA	Missense variant/somatic variant	Heterozygous	4943 G>GA	GAC/AAC	p.Asp50Asn	Asp → Asn	rs776561489

**Table 5:** Illustrates genotypes and allele frequencies for TLR4 exon 3 and MIR155.

Gene	Variant Position	Genotype	Frequency (%)
TLR4 Exon 3	4942 C>CA	CA	14.3%
		CC	85.7%
	4943 G>GA	GA	3.6%
		GG	96.4%
MIR155	12358G>GA	GA	3.6%
		GG	96.4%
	12395T>TC	TC	3.6%
		TT	96.4%

**Table 6:** Shows allele frequencies and genotypes for the MIR155 HG gene.

Variant Position	Genotype	Frequency (%)
12264A>AG	AA	96.4%
	AG	3.6%
12300T>TC	TC	3.6%
	TT	96.4%
12310G>GA	GA	3.6%
	GG	96.4%
12314A>AT	AA	96.4%
	AT	3.6%
12408T>TG	TG	7.1%
	TT	92.9%
12434C>CG	CC	96.4%
	CG	3.6%
12442T>TC	TC	28.6%
	TT	71.4%
12448G>GC	GC	32.1%
	GG	67.9%
12471G>GC	GC	17.9%
	GG	82.1%
12481T>TA	TA	32.1%
	TT	67.9%
12511T>TG	TG	11.5%
	TT	88.5%
12514T>TA	TA	11.5%
	TT	88.5%
12515C>CG	CC	96.2%
	CG	3.8%
12518T>TA	TA	7.7%
	TT	92.3%
12523T>TC	TC	19.2%
	TT	80.8%
12534T>TG	TG	10.0%
	TT	90.0%

**Table 7:** Summary of Mutations of the TLR4 gene retrieved from the gnomAD database.

Gene	Total	Missense Variant	Synonymous Variant	Intron Variant	5_Prime_UTR_Variant	Frame Shift Variant	Stop Gained	3_Prime_UTR_Variant	In- Frame Dele	Splice Region Variant	Start Lost	Splice Acceptor Variant	In- Frame Insert	Splice Donor Variant	Stop Lost
TLR4	1847	1000	412	195	59	55	47	47	11	10	4	4	1	1	1

**Table 8:** Mutation summary of the MIR155HG and miR-155 genes retrieved from the gnomAD database.

Gene	Total	Non-Coding Transcript Exon Variant	Intron Variant	Splice Region Variant	Splice Acceptor Variant	Splice Donor Variant	Mature miRNA Variant	Non-Coding Transcript Exon Variant
MIR155 HG	511	412	84	8	5	2	—	—
miR-155	15	—	—	—	—	—	10	5

**Table 9:** Overview of analytical forecasts on the impact of the TLR4 D50N mutation on the protein stability of the structure.

Prediction Method	Output Type	Predicted Delta\Delta G (kcal/mol)	Effect on Stability	Confidence Score
SVM & Sequence Information	Quantitative	−0.6504	Decrease	N/A
Support Vector Machine	Qualitative	N/A	Decrease	−1.00
Neural Network	Qualitative	N/A	Decrease	−0.76

## Data Availability

The datasets generated and/or analysed during the current study are available in the [NCBI] repository, www.ncbi.nlm.nih.gov/SNP/snp_viewTable.cgi?handle=WISSAM_ALBEER. (Accessed on 31 July 2021).
